# Cryo-EM Reveals VP1/VP2 N-Terminal Conformations in Adeno-Associated Virus 2 Capsids

**DOI:** 10.64898/2026.10.16.759763

**Published:** 2026-10-17

**Authors:** Innocent O. Osuya, Shirin S. Kamalaldinezabadi, Ananya Sengupta, Gwimoon Seo, Michael S. Chapman, Scott M. Stagg

## Abstract

The AAV2 capsid assembles from three viral proteins, VP1, VP2, and VP3, expressed from a single cap gene. In the assembled T=1 60-subunit icosahedral capsid, they have a canonical stoichiometry of 1:1:10 . Relative to VP3, VP1 and VP2 have N-terminal extensions that are required for endosomal escape and nuclear trafficking; however, these regions remain poorly resolved due to low stoichiometry, symmetry mismatches, and conformational heterogeneity of their N-termini. Here, we generated empty AAV2 capsids using a baculovirus expression system with time-course harvests, yielding approximately 2:3:5 and 1:2:7 VP1:VP2:VP3 ratios on days 3 and 5 post-infection, respectively. Cryo-EM single-particle and subparticle reconstructions revealed distinct N-terminal configurations associated with these different VP stoichiometries. Day 3 capsids contained an internal density protruding from the twofold axis and lacked ordered βA preceding residue 237, whereas day 5 capsids lacked the twofold protrusion and instead showed density associated with the fivefold axis, with βA ordered to approximately residues 219 - 221. Fivefold subparticle reconstruction of day 5 capsids further resolved a density plug occluding the pore and an underlying density ring. These findings link VP stoichiometry to distinct conformations of the VP1/VP2 N-termini and provide structural evidence for alternative organization of these regions within the AAV2 capsid, offering new insights into the conformational landscape of AAV capsid heterogeneity.

**Significance:**

Endosomal escape and nuclear trafficking are fundamental to adeno-associated virus (AAV) transduction, yet the structural conformations of the N-terminal regions of the capsid proteins (VP1 and VP2), which are critical for these processes, remain poorly understood because of their low stoichiometric abundance, conformational flexibility, and the limitations of icosahedral symmetry averaging. Using a baculovirus expression system to increase the relative stoichiometric abundance of VP1 and VP2, we applied cryo-electron microscopy with local signal subtraction and focused classification to reveal previously uncharacterized structural features, including distinct βA-strand conformations, an internal density protrusion at the twofold symmetry axis, and differences in the density plug at the fivefold pore. These findings advance our understanding of AAV2 capsid structural heterogeneity and provide a foundation for investigating how VP1/VP2 conformational variability relates to capsid function during infection.

